# Prevalence of benign prostatic hyperplasia among the adult general population of five Middle Eastern Countries: Results of the SNAPSHOT programme

**DOI:** 10.1080/2090598X.2021.2010451

**Published:** 2022-01-23

**Authors:** Amr Noweir, Ashraf Abusamra, Abdelqader Al Zarooni, Murat Binbay, Adam Doble, Luqman Tariq, Fayaz Aziz, Abdelkader El Hasnaoui

**Affiliations:** a Faculty of Medicine, Ain Shams University, Cairo, Egypt; b Division of Urology, Department of Surgery, King Abdul-Aziz Medical City, Jeddah, Saudi Arabia; c Emergency and Surgical Departments, Sheikh Khalifa General Hospital, Umm Al Quwain, United Arab Emirates; d Department of Urology, Haseki Research and Training Hospital, Istanbul, Turkey; e Foxymed, Paris, France; f Medical Department, GlaxoSmithKline, Dubai, United Arab Emirates

**Keywords:** Benign prostatic hyperplasia, prevalence, middle east, snapshot, quality of life, co-morbidity

## Abstract

**Objectives:**

To present data on the prevalence of benign prostatic hyperplasia (BPH) in five Middle Eastern countries (Egypt, Turkey, Kuwait, Saudi Arabia, and the United Arab Emirates; the latter three forming a Gulf cluster).

**Subjects and Methods:**

The SNAPSHOT programme was a multi-country, cross-sectional epidemiological survey conducted by telephone in a random sample of the adult general population. Subjects were considered to have BPH if they fulfilled the screening criteria, based on diagnosis, symptoms, and treatments received in the past 12 months. Current prevalence (last 12 months) was estimated. Association with co-morbidities was investigated via multivariate logistic regressions. Quality of life (QoL) was assessed using the three-level EuroQol five-dimensions questionnaire (EQ-5D-3 L).

**Results:**

In total, 5034 of 33,486 subjects enrolled in the SNAPSHOT programme were men aged ≥50 years. In all, 998 of these men fulfilled the BPH screening criteria. The overall prevalence of BPH ranged from 13.84% (95% confidence interval[CI] 12.3–15.4%) in Turkey, to 23.76% (95% CI 21.8–25.6%) in Egypt, and 23.79% (95% CI 21.2–26.3%) in the Gulf cluster. Co-morbidities occurred more frequently in men with BPH compared to the non-BPH population (57% vs 31%; *P* < 0.001). Principal co-morbidities associated with BPH were cardiovascular, renal, and diabetes mellitus (*P* < 0.001). The men with BPH reported significantly reduced QoL, with lower EQ-5D-3 L utility values (0.8) compared to the male general population (0.9) aged ≥50 years (*P* < 0.001).

**Conclusion:**

The prevalence of BPH in these five Middle Eastern countries ranges from 13.84% to 23.79%. BPH has a negative impact on QoL and is associated with high levels of co-morbid diseases, indicating a need to better understand the management of the disease to reduce the impact on healthcare systems.

## Introduction

Benign prostatic hyperplasia (BPH) with associated LUTSrepresents the fourth most common disease in the male population aged >50 years. A standardised clinical definition of the disease is unavailable, but the disease is associated with significant morbidity due to LUTS, especially the storage (irritative) symptoms of urgency, urgency incontinence, and nocturia. Whilst the disease is not life-threatening, the clinical manifestation of LUTS can reduce a patient’s quality of life (QoL) considerably [1], interfering with daily activities and disrupting sleep [2]. It is widely acknowledged that the prevalence of BPH increases with age [3].

The prevalence of BPH is difficult to determine in general population samples. Many studies have been conducted over the years, but the lack of a standardised clinical definition makes it difficult to conduct large-scale studies that can be compared easily. For example, a study in the Netherlands reported very different prevalence estimates depending on the case definition used to determine a positive screen; ranging from 9% when BPH was defined based on an International Prostate Symptom Score (IPSS)≥8, prostate volume >30 mL, and maximum urinary flow rate<10 mL/s, to 25% when only an IPSS score ≥8 was used [4].

**Figure 1.**
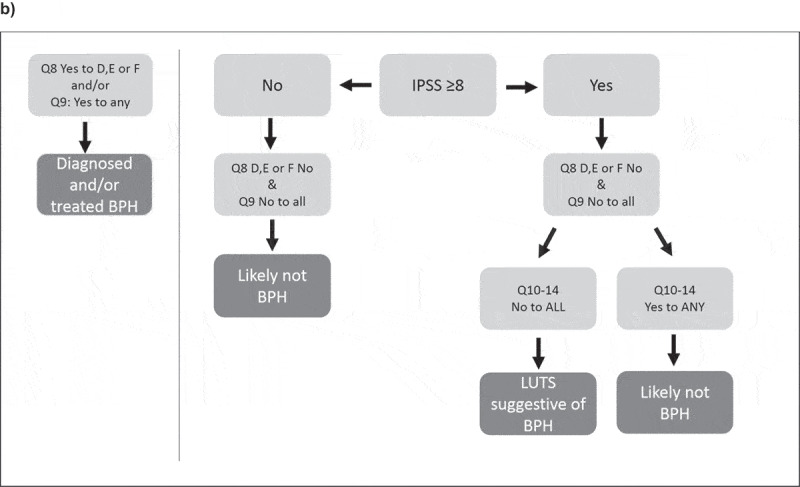
Screening criteria for BPH. (a) Screening questionnaire, (b) Screening algorithm used to determine which subjects are classified as diagnosed and/or treated BPH and LUTS suggestive of BPH.

The meta-analysis included 31 studies, across 25 countries and provided a pooled lifetime BPH prevalence of 26.2% and a median point prevalence of 25.2% [5]. Whilst that study provides a baseline prevalence estimate, the wide variety of study methodologies and case definitions used impacted the results. Most data on the epidemiology of BPH is from North America, Europe, and Asia. There are few studies that have investigated BPH prevalence in the Middle East. Only one study in the recent meta-analysis was conducted in the Middle East, in this case in Saudi Arabia. This was a hospital outpatient survey and reported an overall prevalence of 12% and, as expected, prevalence increased with age [6]. Figure 1

The epidemiology of BPH is poorly defined in the Middle East and there is a need for local data. In order to bridge this gap, the main objectives of the SNAPSHOT BPH study were to assess the prevalence and burden of BPH in five countries within the Middle East and assess the impact of BPH on QoL, using a consistent case definition and study methodology across all countries.

## Subjects and methods

### The SNAPSHOT programme

SNAPSHOT is a cross-sectional, observational, population-based programme, conducted in a random sample of the general population of five countries (Egypt, Kuwait, Saudi Arabia, the United Arab Emirates [UAE], and Turkey) between July 2014 and February 2016. The complete methodology and programme rationale have been described in detail elsewhere [7]. Data from the SNAPSHOT programme on the prevalence and burden of asthma have also been reported [8].

A quota of 10,000 subjects from the adult general population of Turkey and Egypt and 15,000 from the Gulf cluster (Kuwait, Saudi Arabia, and the UAE) was sampled using a random stratified sampling method, based on the demographic structure of the country in terms of age and gender from the most recent census at the time. Additional weight was given to the stratum of men aged ≥50 years. In the Gulf countries, it was difficult to reach the target sample size, and as the combined number of interviews conducted to date in Saudi Arabia, Kuwait and UAE showed that the programme objectives could be achieved from a sample size perspective, recruitment was stopped in February 2016 and the database locked on 11 April 2016.

The programme was carried out by computer-assisted personal interviewing (CAPI) conducted over the telephone, and the information was collected via web-based electronic data capture. Verbal consent to participate was collected and recorded in the CAPI system. The interviewee was then invited to respond to a first questionnaire, screening for the four diseases of interest and documenting social and demographic characteristics, as well as the presence of co-morbidities. If a subject screened positive for at least one of the four diseases, a second disease-specific questionnaire was administered to collect additional information on burden of disease, disease management, and healthcare resource consumption. Should a respondent fulfil the screening criteria for more than one disease, they were randomised by the CAPI system to respond to only one of the disease-specific questionnaires, to limit the duration of the interview. The full interview was conducted in one telephone call. For all subjects, the interview ended with completion of the three level EuroQol five-dimension questionnaire (EQ-5D-3 L) and EuroQol visual analogue scale (EQ-VAS; EuroQol Research Foundation) [9] to collect data on QoL. Trained personnel conducted interviews in Arabic, English or Turkish using translated validated questionnaires.

### Case definition for BPH

Subjects were defined as having BPH based on their IPSS, history of BPH diagnosis or treatment, and differential diagnosis of LUTS. The screening questionnaire comprised of 14 questions in total and was based on that used in the Triumph study [10]. Step one covered completion of questions one–seven of the IPSS, a questionnaire comprising seven questions on urinary symptoms, plus an additional question concerning QoL [11]. Step two comprised two questions asking about a previous diagnosis of BPH and treatment for BPH. Step three involved five questions to enable a differential diagnosis of LUTS, as there are many potential causes of urinary symptoms in men such as diabetes mellitus or Parkinson’s disease [10]. The full screening criteria for BPH are shown in Figure 1.

In order to screen for the disease an algorithm was used to define a positive screen. A subject was defined as ‘diagnosed and/or treated BPH’ if he answered yes to D, E or F in question eight, and/or yes to any of the medications listed in question nine. A subject was defined as ‘LUTS suggestive of BPH’ if he had an IPSS of ≥8 and answered no to questions eight and nine, as well as answered no to questions 10–14. A subject was classified as ‘not likely to have BPH’ if he answered yes to any question from 10 to 14 or had an IPSS of <8 and answered no to questions eight and nine. The overall prevalence of BPH reported here comprises those subjects who fulfilled the criteria of diagnosed and/or treated BPH and LUTS suggestive of BPH. The full screening algorithm is shown in Figure 1.**Figure 1.**
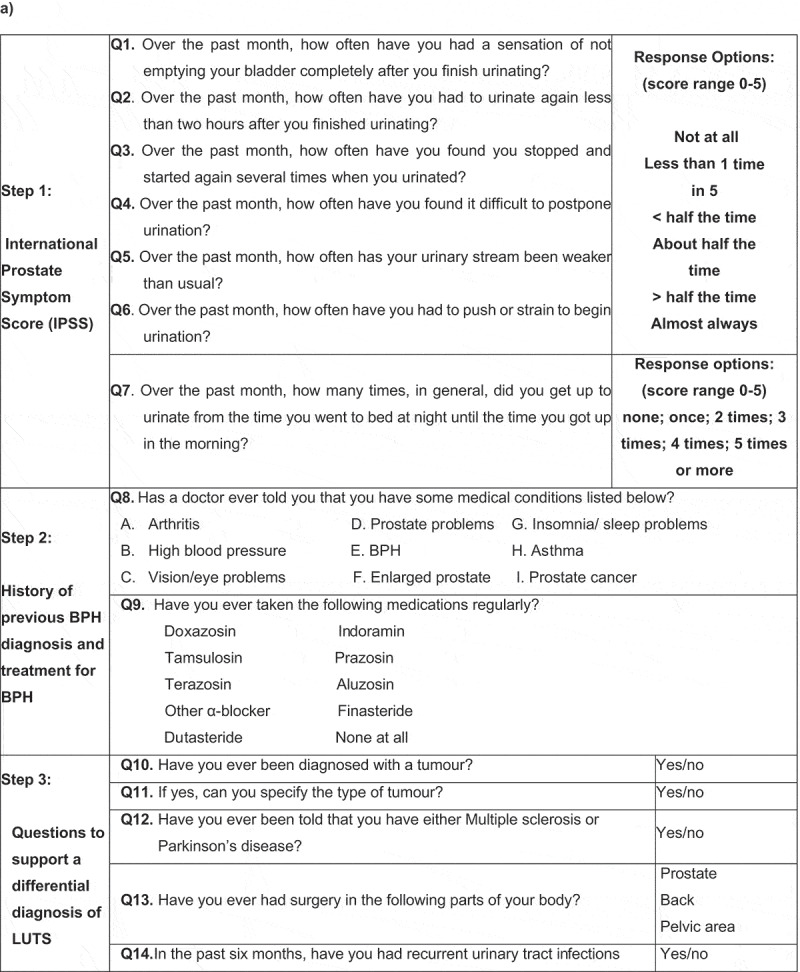
Screening criteria for BPH. (a) Screening questionnaire, (b) Screening algorithm used to determine which subjects are classified as diagnosed and/or treated BPH and LUTS suggestive of BPH.

### Data collected for this analysis

The analysis presented here aimed to provide an estimate of the current prevalence of BPH (overall, diagnosed and/or treated BPH and LUTS suggestive of BPH) in the countries or cluster studied. Therefore, the analysis was restricted to male subjects aged ≥50 years. Sociodemographic data were collected to describe the characteristics of the overall study population, including body mass index (BMI), the presence of co-morbidities, and smoking status. BPH prevalence data were collected using the case definition described and the prevalence by country and region were assessed. All subjects were also asked to complete the EQ-5D-3 L questionnaire to measure QoL, a generic questionnaire to measure health status.

### Statistical analysis

Data are presented as proportions and means with standard deviations (SDs), or medians with interquartile ranges (IQRs). The 95% CIswere calculated for binomial data. Associations between categorical variables were estimated using the chi-square test and the Cochran–Mantel–Haenszel test as appropriate. Two-sided tests were used in all cases and a probability threshold of 0.05 was considered significant. Multivariate regression analysis was performed to assess the relationship between co-morbidities and BPH. All statistical analyses were performed using the Statistical Analysis System (SAS®), version 9.4 (SAS Institute Inc., Cary, NC, USA).

## Results

### Study sample

A total of 5034 male subjects aged ≥50 years agreed to participate in the study, completed the screening questionnaire and thus constituted the screening population. This population was distributed between Egypt (1978), Turkey (2001), and the Gulf cluster (1055). Selected demographics of the screening population are shown in Table 1. These data highlight that a large proportion of the screening population were either overweight or obese (70.7%), just over half were either current or former smokers (55.2%) and almost two-thirds did not report any co-morbidities (64.1%). Almost half the subjects in the Gulf cluster (40.8%) and Egypt (47.3%) had no health insurance, compared to only 6% in Turkey, where the majority are covered by social security.

**Table 1.** Selected demographics of the male subjects aged ≥50 years in the SNAPSHOT screening population (N = 5034)

		Overall N = 5034	Egypt N = 1978	Gulf cluster N = 1055	Turkey N = 2001
BMI, n (%)	Count	4567	1797	938	1832
	Underweight	70 (1.5)	42 (2.3)	10 (1.1)	18 (1)
	Normal weight	1268 (27.8)	492 (27.4)	231 (24.6)	545 (29.7)
	Overweight	1928 (42.2)	677 (37.7)	398 (42.4)	853 (46.6)
	Obese	1301 (28.5)	586 (32.6)	299 (31.9)	416 (22.7)
Smoking-status, n (%)	Count	4873	1903	1015	1955
	Non-smoker	2181 (44.8)	687 (36.1)	532 (52.4)	962 (49.2)
	Smoker or former smoker	2692 (55.2)	1216 (63.9)	483 (47.6)	993 (50.8)
Number of pack-years of cigarettes, n (%)	Count	2655	1205	475	975
	<10 pack-years	533 (20.1)	269 (22.3)	151 (31.8)	113 (11.6)
	≥10 pack-years	2122 (79.9)	936 (77.7)	324 (68.2)	862 (88.4)
Co-morbidities, n (%)	Count	5034	1978	1055	2001
	No	3229 (64.1)	1144 (57.8)	607 (57.5)	1478 (73.9)
	Yes	1805 (35.9)	834 (42.2)	448 (42.5)	523 (26.1)
Health system coverage for consultation and medicines, n (%)	Count	4746	1849	966	1931
	Public	955 (20.1)	730 (39.5)	223 (23.1)	2 (0.1)
	Private/Insured	537 (11.3)	178 (9.6)	310 (32.1)	49 (2.5)
	Social security	1787 (37.7)	18 (1)	11 (1.1)	1758 (91)
	Personal finances	76 (1.6)	47 (2.5)	28 (2.9)	1 (0.1)
	Other	8 (0.2)	2 (0.1)	0	6 (0.3)
	Not insured	1383 (29.1)	874 (47.3)	394 (40.8)	115 (6)

### Current prevalence of BPH

Of the participants enrolled in the study, 998 fulfilled the case definition for BPH and were defined as the BPH population. The adjusted overall prevalence of BPH in the male population aged ≥50 years in the countries studied was 13.8% in Turkey, 23.8% in Egypt, and 23.8% in the Gulf cluster (Table 2). Based on the case definition of BPH used in the SNAPSHOT programme, the BPH population can be sub-categorised into two classes (Table 2); diagnosed and/or treated BPH and LUTS suggestive of BPH.

**Table 2.** Current prevalence of BPH: current prevalence (%) of BPH by country and cluster

	Variable	Egypt	Gulf cluster	Turkey
Overall	Count, N	1978	1055	2001
	Number of cases	470	251	277
	Prevalence, %	23.76	23.79	13.84
	95% CI	21.9–25.6	21.2–26.4	12.3–15.4
	*P*	<0.001		
Diagnosed and/or treated BPH	Number of cases	265	125	219
	Prevalence, %	13.4	11.85	10.9
	95% CI	11.9–14.9	9.9–13.8	9.6–12.3
	*P*	0.058		
LUTS suggestive of BPH	Number of cases	205	126	58
	Prevalence, %	10.4	11.9	2.9
	95% CI	9.0–11.7	10.0–13.9	2.2–3.6]
	*P*	0.001		

*P* values were calculated using the chi square test.

**Figure 2.**
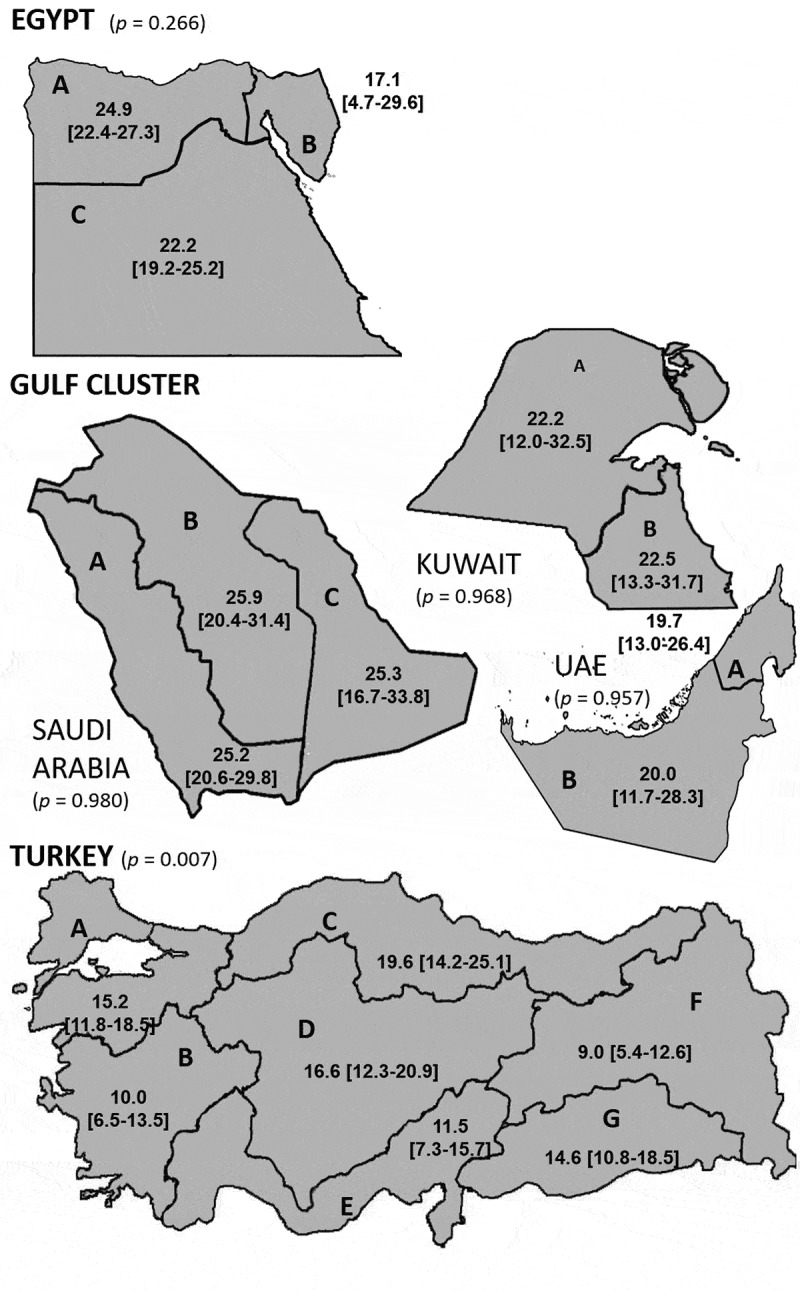
Overall prevalence of BPH by region. Prevalence of BPH (% [95% CI]) by region across the countries studied *P value (chi-square test); Egypt: A = Greater Cairo/North Egypt, B = Canal/other, C = Upper Egypt. Kuwait: A = Western Kuwait, B = Eastern Kuwait. UAE: A = East UAE, B = Abu Dhabi. Saudi Arabia: A = Western Saudi Arabia, B = Central Saudi Arabia, C = Eastern Saudi Arabia. Turkey: A = Marmara, B = Aegean, C = Black Sea, D = Central Anatolia, E = Mediterranean, F = Eastern Anatolia, G = South-eastern Anatolia.

Current overall BPH prevalence was also investigated by region across the countries studied and these results are shown in Figure 2. In Egypt, the highest prevalence was documented in Greater Cairo/North Egypt, and Upper Egypt followed by Canal/Other where the prevalence was lower; however, these differences were not statistically significant (*P* > 0.05). In the Gulf cluster, there was also no significant difference in prevalence across the countries and regions studied (*P* > 0.05). The highest prevalence was reported in Saudi Arabia (25.5%), followed by Kuwait (22.4%), and the UAE (19.7%), but in each country the prevalence was consistent across the country. In Turkey, the highest prevalence was reported in the Black Sea region (19.6%), followed by Central Anatolia (16.6%), Marmara (15.2%), and South-eastern Anatolia (14.6%). The reported prevalence was lower in the Mediterranean (11.5%), Aegean (10.0%) and Eastern Anatolia regions (9.0%) (*P* < 0.05).

### History of co-morbidities and the risk of BPH

**Figure 3.**
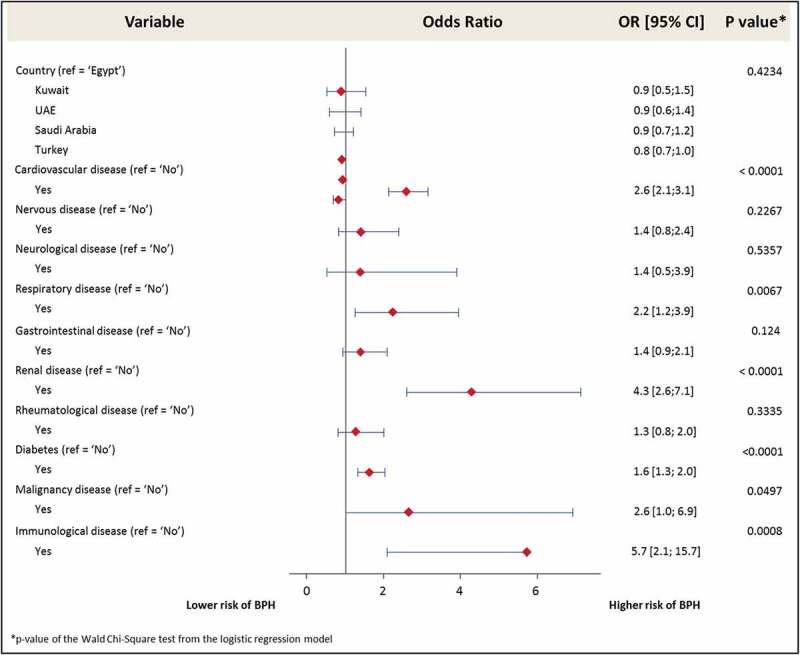
Multivariate regression analysis investigating the association between BPH and co-morbidities. BPH population (609 subjects) vs non-BPH population (4036 subjects.

The number of subjects who reported having a chronic health condition was significantly higher (*P* < 0.001) in the BPH population (56.9%) compared to the non-BPH population (30.7%). In a multivariate regression analysis, the co-morbidities associated with increased risk of BPH (*P* < 0.001) were renal disease (odds ratio [OR] 4.3, 95% CI 2.6–7.1), cardiovascular disease (OR 2.6, 95% CI 2.1–3.1), and diabetes (OR 1.6, 95% CI 1.3–2.0). These results are presented in Figure 3.

### Impact of BPH on QoL

**Figure 4.**
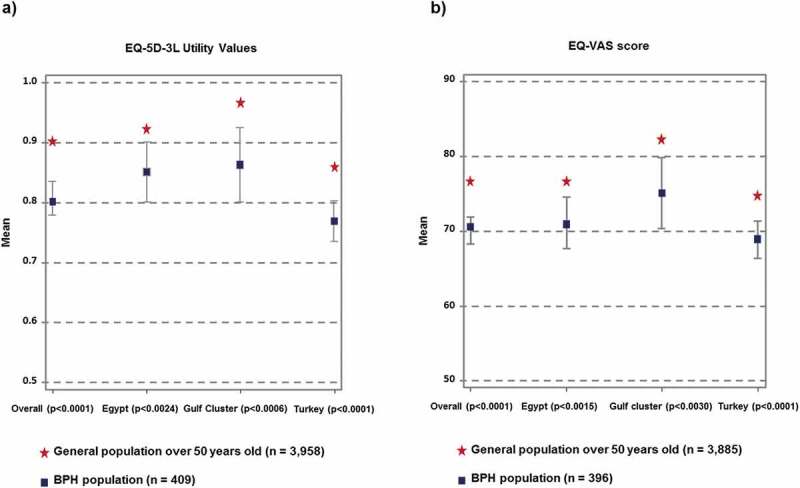
Impact of BPH on QoL. Comparison of the EQ-5D-3 L utility values (a) and EQ-VAS scores (b) between the BPH population and the general population (males aged ≥50 years) by country or cluster. (a) For the BPH population (n = 409) the data represent the mean EQ-5D-3 L utility value with the 95% CI. For the general population (n = 3958), the mean EQ-5D-3 L utility value is presented. (b) For the BPH population (n = 396) the data represent the mean EQ-VAS score with the 95% CI. For the general population (n = 3885), the mean EQ-VAS score is presented.

Overall, subjects with BPH reported a significantly lower (*P* < 0.001) mean EQ-5D-3 L utility score (mean [SD] score 0.80 [0.28]) than the general population of men aged ≥50 years (mean [SD] score 0.90 [0.20]). This relationship was observed for all participating countries or cluster of countries. A similar observation was made for the mean EQ-VAS scores in men with BPH (mean [SD] score 70.37 [18.44]) compared to the general population of men aged ≥50 years (mean [SD] score 76.6 [17.03]) (*P* < 0.001). The overall impact and the country-level results are presented in Figure 4, showing that BPH has a negative impact on QoL.

## Discussion

The primary objective of the present study was to estimate the prevalence of BPH in five countries in the Middle East (Egypt, Turkey, Kuwait, Saudi Arabia, and the UAE) using a consistent case definition and study methodology. The results of the present study show that the overall prevalence of BPH in men aged ≥50 years in these five countries ranged from 13.84% in Turkey to 23.76% in Egypt and 23.79% in the Gulf cluster.

The reported prevalence in SNAPSHOT is significantly lower in Turkey compared to Egypt and the Gulf cluster. There are many possible explanations for this difference, including cultural differences, environmental factors, or genetic factors. The reported prevalence of diagnosed and/or treated BPH is similar across all countries or cluster of countries. However, the prevalence of LUTS suggestive of BPH was only 2.9% in Turkey compared to 10.4% and 11.9% in Egypt and the Gulf cluster, respectively. This might suggest that BPH is identified at an earlier stage of the disease and is generally well-diagnosed in Turkey compared to Egypt and the Gulf countries. Potentially, the underlying mechanism for this difference could be the fact that ~91% of the Turkish subjects had healthcare coverage through social security, whereas in Egypt and the Gulf cluster, many subjects are uninsured (47% and 41%, respectively). This could make healthcare more accessible in Turkey compared to the other countries in which healthcare coverage may be limited, thus enabling subjects in Turkey to visit a physician more frequently and plan more scheduled visits. Another possibility is that subjects with LUTS in Turkey are filtered out due to earlier identification of another competing disease such as prostate or other types of cancer, or neurological diseases.

The algorithm used in the SNAPSHOT programme to screen subjects for BPH was based on the Triumph study [10], a major epidemiological study of LUTS/BPH in primary care in the Netherlands. The overall prevalence of LUTS/BPH reported in the Triumph study was 10.3%, lowest among men aged 45–49 years (2.7%) and increased with age until a maximum at the age of 80 years (24.9%) [10]. This is much lower than the overall prevalence reported in SNAPSHOT in Egypt and the Gulf cluster and similar to that reported in Turkey. However, the Triumph study was conducted using the Primary Care Information database (IPCI), whereas SNAPSHOT was a prospective population-based study. In addition, there may be cultural differences between these countries that could play a role. The Triumph project has conducted similar studies in the UK using the Clinical Practice Research Datalink (CPRD) and THALES in France [12]. Additional Triumph studies have been carried out in six European countries but these studies focus more on the management of LUTS/BPH in general practice [13].

The main risk factors for BPH are thought to be age and hormone levels [14]. In addition, many lifestyle factors have been reported to be associated with BPH, e.g. obesity, physical activity, and diet [14]. Increased adiposity (defined as either increased body weight, BMI, and waist circumference) is known to be correlated with an increased prostate volume, a reliable diagnostic measure of BPH, and obesity has also been shown to increase the risk of LUTS, as measured by the IPSS [14]. Overall, 70.7% of the screening population and 72.7% of the BPH population were overweight or obese, and this was consistent across all countries and could be linked to the prevalence of BPH reported in the present study.

Consistent with the published literature [15], the present study found that chronic conditions, associated with BPH included cardiovascular disease, diabetes, and renal diseases. Given the high incidence of obesity in the screening population, and the age of the subjects, the impact of cardiovascular disease is not unexpected, as obesity and older age both increase the risk of cardiovascular disease. Given the frequent occurrence of these conditions in ageing men, a large proportion of patients can be expected have such an association. In addition, there is a large body of evidence supporting an association between metabolic syndrome, which comprises at least three of the five following features: abdominal obesity, high blood pressure, impaired blood glucose or diabetes, elevated serum triglycerides and reduced high-density lipoprotein, and LUTS [16]. There are numerous reports of an association between diabetes mellitus and BPH, specifically hyperglycaemia and insulin resistance [17]. Chronic kidney disease has also been shown to be associated with BPH [18].

LUTS/BPH is known to have a negative impact on health-related QoL. It has been reported to have an impact on work productivity, social and family life, mental health, and sleep quality. For example, a community-based study in the UK reported QoL (measured using the EQ-5D questionnaire) decreased as LUTS score increased [19]. Nocturia is one of the most bothersome symptoms of BPH and interferes with sleep quality, which can have a very negative effect on a person’s perception of their QoL [20]. In support of the literature, the results from the SNAPSHOT study show that subjects with BPH report a lower QoL than the general population.

It is worth noting that benign prostatic obstruction (BPO) may be considered an alternative term for prostate-related aetiology causing LUTS. However, BPH is still widely used in clinical practice to describe BOO, understanding that this is often caused by benign prostatic enlargement resulting from histological BPH.

The present study has some limitations. The survey was telephone-based, which could introduce a sampling bias. However, both mobile and fixed landline telephone numbers were included in the study, and most households in these countries have access to a mobile telephone. Due to the study design, recall bias may influence data accuracy. In addition, the survey was conducted by trained lay interviewers; therefore, the diagnosis of BPH or other reported co-morbidities was not confirmed by a physician. Although there were no diagnostic tests carried out to confirm the diagnosis, the case definition was designed to take into account that LUTS can be associated with conditions other than BPH and exclude those subjects to provide a differential diagnosis of the symptoms as BPH.

## Conclusion

The SNAPSHOT BPH study provides data on BPH prevalence in five countries in the Middle East, and the use of a standardised case definition and study methodology allows comparisons to be made between the prevalence reported in each country. The reported prevalence of BPH in these five Middle Eastern countries ranges from 13.84% to 23.79%. These prevalence estimates are within the range reported by studies elsewhere in the world that use a similar case definition. In addition, renal disease, cardiovascular disease, and diabetes are associated with an increased risk of BPH, and the disease has a negative impact on QoL. BPH is an age-related disease; globally, populations are ageing, and the number of men diagnosed with BPH is increasing. Therefore, management of the disease is a major challenge for the health system. There is a need to better understand how this burden of disease can be managed to improve QoL for those with BPH, to aid health service planning, and reduce the impact on healthcare systems.

## Abbreviations

BMI: body mass index; CAPI: computer-assisted personal interviewing; CRO: Contract Research Organisation; EQ-5D: EuroQoL five-dimension questionnaire; EQ-5D-3L: three-level EQ-5D questionnaire; EQ-VAS: EuroQol visual analogue scale; GEP: Good Epidemiological Practice; GERD: gastro-oesophageal reflux disease; HTA: health technology assessment; IQR: interquartile range; OR: odds ratio; QoL, quality of life; UAE: United Arab Emirates
